# Sex Differences in the Bitterness Perception of an Aromatic Myrtle Bitter Liqueur and Bitter Compounds

**DOI:** 10.3390/nu15092030

**Published:** 2023-04-23

**Authors:** Antonella Rosa, Ilenia Pinna, Alessandra Piras, Silvia Porcedda, Carla Masala

**Affiliations:** 1 Department of Biomedical Sciences, University of Cagliari, Cittadella Universitaria, SS 554, Km 4.5, 09042 Monserrato, Italy; ilenia.pinna.1994@gmail.com (I.P.); cmasala@unica.it (C.M.); 2 Department of Chemical and Geological Sciences, University of Cagliari, Cittadella Universitaria, SS 554, Km 4.5, 09042 Monserrato, Italy; apiras@unica.it (A.P.); porcedda@unica.it (S.P.)

**Keywords:** bitter perception, quinine, aromatic herbs and plants, bitter liqueur, myrtle

## Abstract

We evaluated sex differences in the perception of bitter compounds and an aromatic bitter herbal liqueur (Mirtamaro) obtained by the infusion of myrtle leaves/berries together with a mixture of Mediterranean herbs/plants as flavoring/bittering ingredients. In a healthy population (*n* = 231 participants), using bivariate correlations and multivariate linear regression analyses, significant sex differences emerged in quinine bitterness perception, with women showing a higher bitter taste intensity rating than men. Among all participants, 40 subjects (subpopulation) were randomly selected for the evaluation of sex differences in Mirtamaro gustatory and olfactory perception using a hedonic Likert-type scale. Women showed higher ratings in Mirtamaro aroma (odor intensity) and bitterness (taste intensity) perception than men, with a superior capacity to perceive/describe its sensory attributes. 1,8-Cineole and methyl chavicol were the main contributors to the bitter liqueur aroma. A significant correlation (r = 0.564, *p* < 0.01) between Mirtamaro odor pleasantness/taste pleasantness was observed in women, indicating a positive contribution of aromatic herbs to bitter taste acceptability. Moreover, a higher bitter intensity rating of 6-n-propylthiouracil was evidenced in women than men. Our results highlighted sex differences in bitter taste acuity and the role of aromatic herbs/plants in modulating bitter taste acceptance, which is useful information in the field of precision nutrition and medicine.

## 1. Introduction

The bitter taste is one of the basic taste modalities and bitterness perceptions, often associated with food aversions and rejection behavior, and is considered a defense mechanism against harmful compounds (toxicants) [1,2,3,4]. Plants produce bitter toxic substances as a defense strategy against herbivores [3,4]. However, numerous natural bitter compounds have negligible toxicity and exert important health benefits (antioxidant and anti-inflammatory properties) [1,4,5]. Indeed, several herbs rich in bitter chemicals are often used in traditional Chinese and Ayurveda medicines for their beneficial properties [1,3,5,6]. The receptors for bitter taste are called taste 2 receptors (TAS2Rs or T2Rs) and are a subfamily of G protein-coupled Receptors (GPCRs) on the taste bud cells of the tongue and oral cavity [1,3,5,7,8]. Bitter taste receptors, TAS2Rs, expressed in extraoral tissues may also be responsible for some physiological effects exerted by bitter compounds and are thus considered potential drug targets for the treatment of several diseases and disorders [5,8,9].

The consumer’s perception of bitter taste is a key factor in the acceptability and success of foods and drugs; therefore, food/pharmaceutical industries aim to mask or minimize bitterness to increase the acceptance of food products and avoid treatment refusal [2,4,6,7]. However, bitter substances can be well tolerated, and certain bitter foods (chocolate, broccoli, and whole wheat bread) and beverages (coffee and tea) are known to be safe for consumption [1,2,4]. It has been demonstrated that a moderate amount of bitterness may enrich flavor and confer physiological functions on alcoholic beverages [2,10,11,12]. Indeed, bitterness is a basic flavor in beer, wine, herbal liquors, and rice wine [2,10,11,12]. Bitter compounds such as caffeine, polyphenols, glucosinolates, and humulones have health benefits in the concentrations at which they are typically consumed [1,2,4]. Therefore, removing or breaking down healthy bitter phytonutrients may reduce the food’s beneficial effects [2,4].

The bitter taste is also constantly dropping its popularity among consumers [2,4,13]. The consumption of bitter herbal liqueurs has greatly increased in the last years due to their digestive and tonic-restorative properties [10,11,12,13,14,15,16]. Italy possesses a great tradition in the preparation of “Amari” [11,12], alcoholic (above 15°) aromatic preparations with a distinctly bitter taste, used as an aperitif to stimulate the appetite or consumed after a meal to aid food digestion (eupeptic properties) [10,11,12,13,14,15,16]. Moreover, bitter herbal liqueurs generally possess considerable antioxidant properties due to their high polyphenol content [10,11,12,14,15]. Traditional Italian bitters are made by the infusion/maceration of a mix of different parts (barks, rhizomes, roots, berries, flowers, and peels) of selected aromatic and bitter herbs/spices/plants in a hydroethanolic base [11,12]. Herbal bitter liqueurs’ formulation requires the blending of bitter-tasting herbs/plants (generally *Gentiana*, *Artemisia*, and *Achillea* spp.) with aromatic ones, with one being the main contributor to the final sensory properties of the product, whereas others are used for flavor or color corrections [10,11].

Gustatory perception of the bitter taste is a promising area of study because of its role in food choices, feeding behavior, and food’s perceived healthiness [4,17,18,19]. Many studies are focused on the investigation of the genetic [1,2,7,9,17] and age [17,18,19] differences regarding bitterness perception; however, there is still limited and heterogeneous information on the influence of sex on bitter taste perception [7,17,18,19]. The knowledge of sex differences in bitter taste acuity has potential applications in precision nutrition/medicine [18,20]. Moreover, strategies are currently required to successfully reduce/mask bitterness in food/pharmaceutical products and increase their acceptance [4,6,7]. There is a great interest in the dietary use of aromatic herbs and spices for their ability to impart distinctive flavorings to food products, contemporaneously increasing their nutritional value and positively affecting human health [21]. Volatile constituents (terpenes and terpenoids) contribute to the flavor and aroma of aromatic herbs and spices [10,11,13]. It has been reported that the rich aromatic substances in alcoholic beverages may influence bitterness perception [2]. Volatile organic flavor compounds are responsible for food/liqueur aroma and are perceived through retro-nasal olfaction [11,12]. It has been evidenced that odor–taste interactions can result in cross-modal summation [22] and that bitterness is cognitively related to aromas [2]. Individual differences largely influence aroma perception [22] and previous studies reported that women exhibit better olfactory ability compared to men [23].

Therefore, starting from all these considerations, the objectives of the present research were to explore the occurrence of sex differences in the perception of bitter compounds and an aromatic bitter herbal liqueur, and to evaluate the modulatory effect of aromatic substances on sex-specific bitterness perception/acceptance. To the best of our knowledge, no previous work has been reported on sex differences in sensory perception of a complex aromatic herbal bitter liqueur.

We initially evaluated the role of sex in the bitter intensity rating of quinine, a common bitter substance [7,21,24] in a healthy population (*n* = 231 Caucasian European participants). Bitterness perception was determined by the “Taste Strips” test [21,25,26]. The correlation between bitter perception with demographic features, other basic tastes (sweet, salty, and sour), and olfactory function was also determined.

Then, in the second part of the study, we evaluated in healthy subjects the sensory perception (odor and taste) and acceptance of a commercial Italian “Amaro”, a bitter aromatic herbal liqueur, to evidence the influence of sex on the bitterness intensity rating and to evaluate whether the presence of volatiles (aroma) from aromatic herbs/plants could influence bitter taste perception/acceptance. The odor and taste perception of the aromatic myrtle bitter liqueur (Mirtamaro) was assessed in a group (subpopulation) of non-trained subjects (*n* = 40). Differences in sensory perception between men and women were evaluated considering the rate of the gustatory and olfactory dimensions of pleasantness, intensity, and familiarity using a hedonic scale method (Likert scale), as previously used for the determination of the sensory properties of food products [21,25,27]. Bitter liqueur was obtained by the maceration of myrtle (*Myrtus communis*) leaves and berries together with a complex mixture of Mediterranean herbs and plants as flavoring/bittering ingredients. Myrtle is an after-meal liqueur typical of Sardinia (Italy), greatly appreciated for its bitter flavor and special aroma, and its tonic, digestive, antioxidant, and anti-inflammatory properties [11,28,29]. Quantitative analyses by gas chromatography–flame ionization detection–mass spectrometry (GC-FID/MS) of the main volatile compounds extracted from Mirtamaro were performed and their potential contribution to bitter perception was evaluated. Moreover, the influence of sex on the intensity rating of the 6-n-propylthiouracil (PROP) was also evaluated in this selected group of subjects.

## 2. Materials and Methods

### 2.1. Chemicals

6-n-Propylthiouracil (PROP; purity ≥ 99%), used for sensory assessment, and *n*-hexane (99.9%, analytical grade) for the extraction of volatile compounds were purchased from Sigma-Aldrich (St. Louis, MO, USA).

### 2.2. Participants

Two hundred and thirty-one subjects were enrolled with an age range of 19–85 years (mean age ± SD, 35.8 ± 15.9), 153 women and 89 men. All subjects received an explanatory statement and gave their written informed consent to participate in the research study. Exclusion criteria were cognitive impairment, head or neck trauma, stroke, chronic/acute rhinosinusitis, neurodegenerative disorders, psychiatric conditions, and any disorder that may interfere with the olfactory and gustatory evaluations, as previously reported [21,25,27]. None of the participants had taken medications (for allergies or other diseases) for 5 days before the test. Age (years), weight (kg), height (m), and body mass index (BMI) were collected for all participants. This study was approved by the “Azienda Ospedaliera Universitaria di Cagliari” Ethical Committee (protocol number: PG/2018/10157) and was performed according to the Declaration of Helsinki.

### 2.3. Gustatory Perception Assessment

The gustatory perception was assessed in all subjects using the “Taste Strips” test (Taste Strips 50 LA-13-00314 test, Burghart Messtechnik, Wedel, Germany). The test consists of filter paper strips (with a length of 8 cm and a tip area of 2 cm^2^) impregnated with four concentrations of each basic taste quality: sweet, bitter, sour, and salty [21,26]. Concentrations were: 0.4, 0.2, 0.1, 0.05 g/mL of sucrose for sweet taste; 0.006, 0.0024, 0.0009, 0.0004 g/mL of quinine hydrochloride for bitter taste; 0.3, 0.165, 0.09, 0.05 g/mL of citric acid for sour; 0.25, 0.1, 0.04, 0.016 g/mL of sodium chloride for salty taste [21,26]. Drinking water was used as the solvent in each taste modality. Before the test, participants rinsed their mouths with drinking water. The global taste score may range from 0 to 16 and a score ≥ 9 is considered normogeusia [21,26].

### 2.4. Olfactory Function Assessment

The olfactory function was assessed using the Sniffin’ Sticks test (Burghart Messtechnik, Wedel, Germany), which consists of three different tasks, odor threshold (OThr), odor discrimination (ODi), and odor identification (OId) [25,30,31]. All subjects were allowed to drink only water 1 h before the test and had to avoid smoking and scented products on the testing day. Sniffin’ Sticks consists of pen-like odor-dispensing devices. All participants were blindfolded for the OThr and ODi tasks. Primarily, OThr task was evaluated using 16 stepwise dilutions of n-butanol [25,30,31]. OThr task was assessed employing a three-alternative forced-choice task (3AFC) and single-staircase technique [31]. OThr scores may vary from 16 (subject who could perceive the lowest concentration) to 1 (subject who could not perceive the highest concentration). Secondly, ODi test was assessed over 16 pen-like odor-dispensing devices. In the ODi task, three different pens were used, two containing the same odor and the third containing the target one with the 3AFC task. The ODi total score is calculated as the sum of correct answers and may range from 0 to 16 [25,30,31]. Finally, OId test was evaluated by 16 common odors with four verbal descriptors and a multiple forced choice format (three distractors and one target) [25]. The total olfactory function (TDI score = values of OThr + OId + ODi) was calculated and values over 30.5 indicated normosmia [25,30,31].

### 2.5. PROP Bitterness Assessment

Among all participants. a group of subjects (*n* = 40, 14 men and 26 women) were enrolled to assess the sensory properties of n-6 propylthiouracil (PROP). The PROP bitter taste intensity was performed using three different solutions (0.032. 0.32, and 3.2 mM) according to the literature [32]. Drinking water was used as the solvent and to rinse the mouth. Filter paper strips (4 cm^2^) were soaked in PROP solutions and presented to participants at room temperature. Subjects placed filter paper strips on their tongue and were asked to describe the taste on their tongue. If they reported that perceived bitter taste, the first stimulus was the lowest concentration. If the participant’s first response was tasteless, subsequent stimuli increased in concentration. The PROP bitter intensity score is calculated as the sum of correct answers and may range from 0 to 3.

### 2.6. Aromatic Myrtle Herbal Liqueur

**Figure 1:**
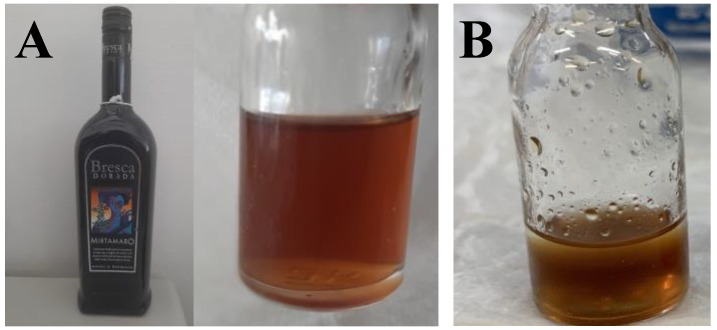
(**A**) Digital image of aromatic myrtle herbal liqueur (Mirtamaro). (**B**) Extraction of Mirtamaro with *n*-hexane.

The aromatic myrtle bitter liqueur (Mirtamaro) (Figure 1A) was produced and kindly provided by the “Bresca Dorada s.r.l.” company (located in Muravera, CA, Sardinia, Italy).

As indicated on the commercial label, the bitter liqueur is obtained by extensive infusion in a hydroethanolic base of myrtle (*Myrtus communis*) together with a complex mixture of aromatic and medicinal Mediterranean herbs and plants as flavoring/bittering ingredients. More than twenty aromatic herbs/plants (gentian root, *Citrus* fruits, licorice, helichrysum, and fennel among others) are mixed with myrtle leaves and berries according to a secret recipe to obtain the right balance of bitter, balsamic, spicy, and citrus flavors of this digestive liqueur. Both wild and cultivated (from organic production on a local farm) herbs/spices and plants are used for bitter liqueur preparation. Herbs and plant parts are used both fresh and after drying. The final alcohol concentration is 30% (*v*/*v*). Another ingredient reported on the label is sugar, added to balance the taste and make it more palatable.

### 2.7. Procedures to Assess Odor and Taste Pleasantness, Intensity, and Familiarity of Aromatic Myrtle Herbal Liqueur (Mirtamaro)

Among all participants, a group of subjects (14 men and 26 women) were randomly enrolled to assess the sensory properties of the aromatic myrtle herbal liqueur (Mirtamaro). Non-trained subjects were asked to evaluate the odor and taste dimensions (pleasantness, intensity, and familiarity) of the bitter liqueur by using a hedonic scale method (self-reported Likert scale) [21,25,27]. Before the sensory assessment, myrtle bitter liqueur was aliquoted, at room temperature (23 °C), in 2 mL disposable plastic test tubes. Initially, the sample was smelled by the subjects, and they were asked to indicate the subjective aroma attributes/descriptors that they perceived with more intensity. Then, participants evaluated the odor pleasantness, intensity, and familiarity of Mirtamaro.

Filter paper strips impregnated with the myrtle bitter liqueur for taste assessment were prepared by immersing the strip in an aliquot (2 mL) of Mirtamaro and removing the alcohol by strip shaking. Before the taste experiment, participants rinsed their mouths with drinking water. Participants evaluated the taste pleasantness, intensity, and familiarity of Mirtamaro and generated subjective sensory attributes, such as the presence of a particular flavor note or aftertaste. The odor and taste pleasantness, intensity, and familiarity of Mirtamaro were evaluated using a 7-point Likert-type scale, which ranged from 0—not at all to 6 (such as 0 = very unpleasant and 6 = very pleasant; 0 = not intense at all and 6 = very intense; 0 = not familiar at all and 6 = very familiar). A value of 3 was considered a neutral point [21,25,27].

### 2.8. Preparation of n-Hexane Extracts from Aromatic Myrtle Herbal Liqueur (Mirtamaro)

Liquid/liquid extraction, using n-hexane as the solvent, was used to extract volatile compounds from Mirtamaro. This non-polar organic solvent is well recognized as suitable for the extraction of a wide class of flavor compounds [21,33]. Briefly, aliquots (3 mL) of the bitter liqueur were treated with 1 mL of n-hexane in a glass screw-cap vial. After 72 h at 25 °C in the dark, the n-hexane supernatant was filtered through a 0.45 μm syringe filter into a vial. Aliquots (1 μL) of n-hexane extracts were directly injected (to avoid volatile compound losses) into the GC/MS system for the determination of the quali-quantitative composition of the main volatile components.

### 2.9. GC-FID and GC-MS Analysis of n-Hexane Extracts from Aromatic Myrtle Herbal Liqueur (Mirtamaro)

Quantitative analyses were performed on a gas chromatograph Agilent 7890A GC (Agilent Technologies, Palo Alto, CA, USA) equipped with a flame ionization detector (FID) and a 30 m × 0.25 mm i.d. with a 0.25 μm stationary film thickness HP-5ms capillary column (Agilent J&W, Palo Alto, CA, USA), coupled with a mass selective detector with an electron ionization device (EI) and a quadrupole analyzer (Agilent 5973) as previously reported [21,34]. The following temperature program was used: from 60 °C to 246 °C at a rate of 3 °C/min and then held at 246 °C for 20 min (total analysis time 82 min). Other operating conditions were the following: carrier gas helium (purity ≥ 99.9999%—Air Liquide Italy); flow rate, 1.0 mL/min; injector temperature, 250 °C; detector temperature, 300 °C. Injection of 1 μL of diluted sample was performed with 1:20 split ratio, using an autosampler (Agilent, Model 7683B). The MS conditions were as follows: MS transfer line temperature 240 °C; EI ion source temperature, 200 °C with ionization energy of 70 eV; quadrupole temperature 150 °C; scan rate, 3.2 scan/s at *m*/*z* scan range, (30 to 480). To handle and process chromatograms and mass spectra, the software Agilent MSD ChemStation E.01.00.237 (Agilent Technologies) was used. Compounds were identified by comparison of their mass spectra with those of NIST02 library data of the GC/MS system [35] and Adams libraries spectra [36]. The results were further confirmed by comparison with the compounds’ elution order with their retention indices on semi-polar phases reported in the literature [35]. Retention indices of the components were determined relative to the retention times of a series of n-alkanes (two standard mix: C_8_–C_20_ and C_21_–C_40_) with linear interpolation [37]. Quantification of constituents was made by integration of GC-FID peak areas without using the response correction factors. The individual volatile compound concentration was expressed as the percentage of the total amount of volatile compounds (% *w*/*w*). Three replicates were performed for each sample.

### 2.10. Statistical Analyses

Data were expressed as a mean ± standard deviation (SD). The evaluation of the statistically significant differences was performed using Graph Pad INSTAT 3.0 software (GraphPad Software, San Diego, CA, USA) and with the software package IBM SPSS Statistics 25 for Windows (IBM, Armonk, NY, USA). The normal distribution of data was calculated using the Shapiro–Wilk test. Statistically significant differences between men and women were performed using Student’s unpaired *t*-test with Welch’s correction, which does not assume those two populations have the same standard deviation. Bivariate correlations using Pearson’s coefficient (r) were calculated between different factors in total subjects, men, and women to identify the more promising factors for the multivariate linear regression analyses. Moreover, the multivariate linear regression analysis with a stepwise selection was performed to assess: the potential contribution of sex, age, weight, and sweet taste perception (independent variables) to the bitter (quinine) taste intensity score (dependent variable) in all subjects, men, and women; the potential contribution of Mirtamaro odor pleasantness, odor intensity, odor familiarity, taste intensity, and taste familiarity dimensions (independent variables) to bitter liqueur taste pleasantness (dependent variable) in men and women. The significance level was set at *p* < 0.05.

## 3. Results

### 3.1. Determination of Olfactory and Gustatory Perception in Subjects

In the first part of the study, we explored the occurrence of sex differences in the olfactory and gustatory perception in a healthy population (Caucasian European participants), specifically focusing our analyses on bitterness perception.

Table 1 indicated mean values ± standard deviation (SD) determined for age, weight, height, BMI, odor threshold (OThr), odor discrimination (ODi), odor identification (OId), and TDI score (OThr + ODi + OId score) measured in total subjects (*n* = 231), men (*n* = 78), and women (*n* = 153).

The mean overall age was 35.8 years, without statistically significant differences between men and women. The age range was from 18 to 85 years old. Significant differences were observed between men and women for the mean weight (*p* < 0.001) and BMI (*p* < 0.001), while similar values were measured for OThr, ODi, OId, and TDI.

Taste perception was determined by challenging subjects with four increasing concentrations of the five basic tastes using sucrose, NaCl, quinine hydrochloride, and citric acid (“Taste Strips” test).

**Figure 2:**
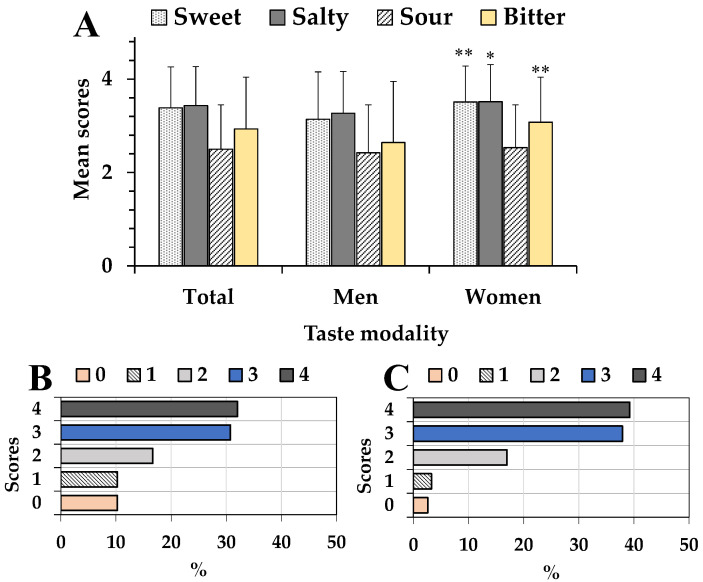
Mean values ± standard deviation of sweet, salty, sour, and bitter (quinine) taste scores measured in total subjects (Total, *n* = 231), men (*n* = 78), and women (*n* = 153). For each taste modality (sweet, salty, sour, and bitter): * = *p* < 0.05, ** = *p* < 0.01 for men versus women (Student’s unpaired *t*-test with Welch’s correction) (**A**). Patterns of subjects’ bitterness perception, expressed as percentual values (%), of each bitter (quinine) taste score (0, 1, 2, 3, and 4), assessed in men (**B**) and women (**C**).

Figure 2A shows mean values ± SD of sweet, salty, sour, and bitter (quinine) taste scores measured in total subjects (Total, *n* = 231), men (*n* = 78), and women (*n =* 153). The percentual values (%) of each bitter (quinine) taste score (0, 1, 2, 3, and 4) determined for men and women are reported in Figure 2B and Figure 2C, respectively.

Values of the intensity rating for the five basic tastes in total participants were 3.4 ± 0.9, 3.4 ± 0.8, 2.6 ± 0.9, 2.9 ± 1.1, and 12.2 ± 2.3 for sweet, salty, sour, bitter (quinine), and total taste scores, respectively.

Significant differences were observed for sweet (*p* < 0.01) and salty (*p* < 0.05) scores between men versus women, which showed the highest intensity rating, while similar values were observed for the sour taste in the two groups. Interestingly, women showed significantly (*p* < 0.01) higher mean values (3.08 ± 0.90) of bitter (quinine) taste intensity ratings than men (2.64 ± 1.31). The patterns of subjects’ bitterness perception, expressed as percentual values (%) of each bitter (quinine) taste score, revealed a preponderance of 3 (37.9%) and 4 (39.2%) score values in women, with low percentages of 0 (2.6%) and 1 (3.3%) scores, whereas men showed lower percentages of 3 (30.8%) and 4 (32.0%) scores and higher percentages of 0 (10.3%) and 1 (10.3%) values than women. Significant differences (*p* < 0.05) in bitter (quinine) perception were also observed between men and women separated into three different age groups [18]: 18–36 years (*n* = 50 for men and *n* = 91 for women), 37–50 years (*n* = 10 for men and *n* = 24 for women), and 51–85 years (*n* = 18 for men and *n* = 38 for women), with women showing the highest intensity rating (Figure S1, Supporting Information).

For the total taste score, as the sum of intensity ratings of the five stimuli, women perceived taste as significantly more intense (*p* < 0.001) than men.

Significant differences (*p* < 0.001) were also observed for bitter taste (quinine) intensity rating between women with a body weight ≤ 65 kg (3.25 ± 0.86) versus those with a body weight > 65 kg (2.42 ± 1.09), indicating a decreased bitterness perception with weight increase (Figure S2, Supporting Information). No statistically significant differences were measured between men and women for sweet, salty, and sour concerning weight.

To evaluate the potential role of sex on bitter (quinine) taste perception, bivariate correlations and multiple linear regression analyses were performed. The relation between bitterness perception versus sex, age, weight, BMI, olfactory, and other gustatory parameters was determined. Table 2 shows Pearson’s correlations and significance measured between bitter (quinine) taste intensity rating versus other parameters in total subjects, men, and women.

Considering all subjects, low significant correlations emerged between bitter taste intensity versus sex, weight, BMI, and sweet taste intensity, while no significant correlations were found versus other parameters. Low significant correlations were found between bitter (quinine) taste intensity versus sweet taste intensity in men, and versus weight and BMI in women (Table 2). The bitter intensity was significantly correlated with total taste perception in all three groups.

Furthermore, the multivariate linear regression analysis using a stepwise selection was performed to assess the potential contribution of sex, age, weight, and sweet taste perception on the bitter (quinine) taste intensity in all subjects. In the multivariate linear regression analysis, the bitter taste intensity was considered the dependent variable, while age, sex, weight, and sweet taste perception were independent variables based on bivariate correlation results. The model was corrected for age unless not significantly correlated with bitter taste intensity since the gustatory function usually decreases with age.

Considering total subjects, the multivariate linear regression analyses (Table 3) showed that sex and sweet taste were significantly associated with the bitter (quinine) taste intensity score (F_(4.230)_ = 6.243, *p* < 0.01) and the model explained around 4% of the variance (R^2^ = 0.035).

In men, significant associations were observed between bitter (quinine) taste intensity score versus sweet taste perception (F_(3.77)_ =2.735, *p* < 0.05). This model explained around 32% of the variance (R^2^ = 0.316).

Instead, in women, the multivariate linear regression analysis showed significant associations between bitter taste intensity score versus weight (F_(3.152)_ = 3.223, *p* < 0.01) with a model that explained around 6% of the variance (R^2^ = 0.061).

### 3.2. Ratings of Odor and Taste Pleasantness, Intensity, and Familiarity of the Aromatic Myrtle Herbal Liqueur (Mirtamaro)

Then, in the second part of the study, we evaluated in healthy subjects the influence of sex on the bitterness perception of the bitter aromatic herbal liqueur Mirtamaro and explored the effect of volatile compounds (aroma) from aromatic herbs/plants on bitter taste perception/acceptance.

Among all participants, a group of subjects (subpopulation, *n* = 40, 14 men and 26 women), with an age range of 21–71 years (mean age of 45.9 ± 19.8), were enrolled to assess the sensory properties of the aromatic myrtle herbal liqueur.

Demographic and clinical features of the total subjects, men, and women are reported in Supporting Information (Table S1). Mean values ± SD of sweet, salty, and sour taste scores measured for total subjects, men, and women are reported in Figure S3 Supporting Information. No significant differences were observed between men versus women for all analyzed parameters, except for weight.

**Figure 3:**
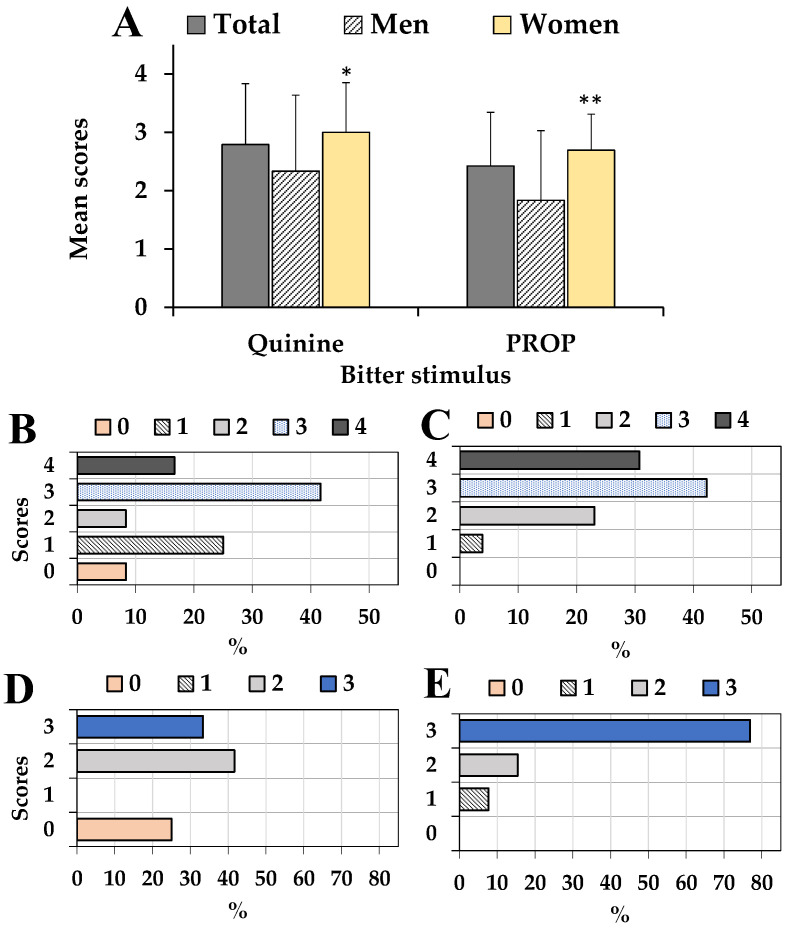
Mean values ± standard deviation (SD) of quinine and PROP taste scores measured in total subjects (Total, *n* = 40), men (*n* = 14), and women (*n* = 26); ** = *p* < 0.01, * = *p* < 0.05 for men versus women (Student’s unpaired *t*-test with Welch’s correction) (**A**). Patterns of subjects’ bitterness perception, expressed as percentual values (%) of each quinine (0, 1, 2, 3, and 4) and PROP (0, 1, 2, and 3) taste score, determined for quinine in men (**B**) and women (**C**) and for PROP in men (**D**) and women (**E**).

In this subpopulation, the intensity rating of the bitter compounds PROP was also determined and compared with the intensity scores obtained for quinine. Figure 3A shows mean values ± SD of the intensity rating of the bitter compounds quinine and PROP measured for total subjects, men, and women. The % values of each quinine taste score (0, 1, 2, 3, and 4) determined for men and women are reported in Figure 3B and Figure 3C, respectively, while Figure 3D and Figure 3E show the % values of each PROP taste score (0, 1, 2, and 3) measured in men and women, respectively.

Moreover, in this subpopulation, women showed a significantly (*p* < 0.05) higher mean value (3.00 ± 0.85) of the quinine bitter taste intensity rating than men (2.33 ± 1.30), with an elevated percentage of 2 (23.1%), 3 (42.3%), and 4 (30.8%) scores.

This trend was also confirmed using PROP as the bitter stimuli, with a significantly (*p* < 0.01) higher intensity perception of bitter taste in women (2.69 ± 0.62) than in men (1.83 ± 1.19). Among women, 76.9% of them perceived all PROP-tested concentrations, while a lower value (33.3%) was observed in men.

Then, non-trained subjects were asked to evaluate the odor and taste dimensions (pleasantness, intensity, and familiarity) of Mirtamaro by using a hedonic Likert-type scale [21,25,27] to evidence the potential role of sex in the perception of bitterness and the possible modulatory effect of aromatic compounds in bitter taste perception.

Participants were initially asked to provide a free description of the odor subjective sensory properties (aroma) of the commercial aromatic bitter liqueur and the results are listed in Table 4.

Regarding Mirtamaro’s odor (aroma), both men and women individuated the presence of alcohol, myrtle extract, and bitter compounds. In addition to myrtle, the main contributor to the sensory properties of the product, participants (untrained panelists) generally had difficulties individuating specific components (herbs/spices) of the flavoring mixture. However, women gave more sensory descriptors than men, in terms of the presence of specific aromas (such as licorice, juniper, berries, orange, woody, etc.).

**Figure 4:**
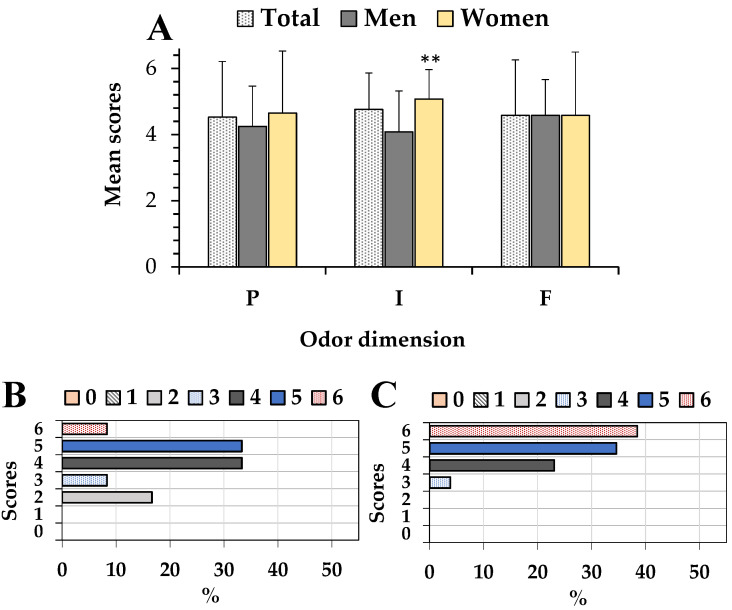
Ratings of odor pleasantness (P), intensity (I), and familiarity (F) dimensions of the aromatic myrtle herbal liqueur (Mirtamaro) measured in total subjects (Total, *n* = 40), men (*n* = 14), and women (*n* = 26). Data are presented as mean values and standard deviations; ** = *p* < 0.01 for men versus women (Student’s unpaired *t*-test with Welch’s correction) (**A**). Patterns of subjects’ odor intensity perception of Mirtamaro, expressed as percentual values (%) of each odor intensity score (0, 1, 2, 3, 4, 5, and 6), determined for men (**B**) and women (**C**).

Figure 4 shows the ratings of odor pleasantness, intensity, and familiarity dimensions of the bitter liqueur measured in total subjects, men, and women (Figure 4A) and the % values of each odor intensity score (0, 1, 2, 3, 4, 5, and 6), assessed in men (Figure 4B) and women (Figure 4C).

In general, high mean scores (>4) were measured both in men and women for all odor dimensions. No significant marked sex differences were observed in the perception of Mirtamaro odor pleasantness and familiarity dimension; however, women perceived Mirtamaro odor as more pleasant than men. Interestingly, a significantly (*p* < 0.01) higher rating in odor intensity was observed in women than men, with 73.1% of women showing perception scores of 5 and 6.

Regarding the taste subjective sensory properties (flavor) of Mirtamaro (Table 4), both men and women indicated bitterness as the main taste perception (the attributes were very bitter or bitter). In general, the time necessary for bitterness perception in the mouth was greater compared with the other taste modalities (sweet, sour), and in many cases, participants indicated an initial sweet taste perception followed by a bitter taste. The occurrence of alcohol and myrtle extract was recognized by both groups. As observed for odor, in the description of Mirtamaro taste perceived attributes, women gave more sensory descriptors than men, in terms of the presence of another specific flavor such as a *Citrus* note, woody note, mint aftertaste, or caramel aftertaste.

**Figure 5:**
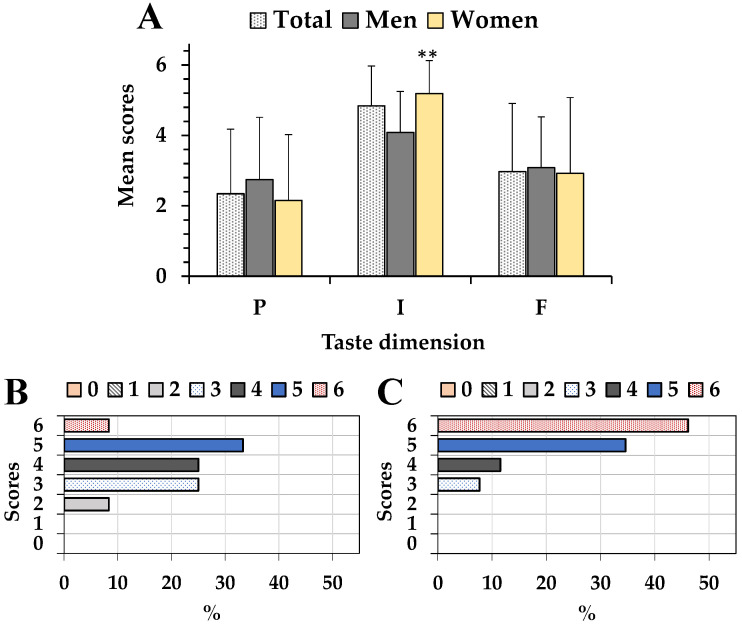
Ratings of taste pleasantness (P), intensity (I), and familiarity (F) dimensions of the aromatic myrtle herbal liqueur (Mirtamaro) measured in total subjects (Total, *n* = 40), men (*n* = 14), and women (*n* = 26). Data are presented as mean values and standard deviations; ** = *p* < 0.01 for men versus women (Student’s unpaired *t*-test with Welch’s correction) (**A**). Patterns of subjects’ taste intensity perception of Mirtamaro expressed as percentual values (%) of each taste intensity score (0, 1, 2, 3, 4, 5, and 6), determined for men (**B**) and women (**C**).

Figure 5 shows the ratings of pleasantness, intensity, and familiarity dimensions of Mirtamaro taste measured in total subjects, men, and women (Figure 5A) and the % values of each taste intensity score (0, 1, 2, 3, 4, 5, and 6), determined in men (Figure 5B) and women (Figure 5C).

In general, both men and women showed lower ratings of taste pleasantness and familiarity than the same odor dimensions. As observed for odor, no significant sex differences were observed in the perception of Mirtamaro taste familiarity. Regarding pleasantness, women perceived the bitter liqueur taste as less pleasant (2.15 ± 1.87) than men (2.75 ± 1.76). Moreover, a significantly (*p* < 0.01) higher rating in taste intensity was observed in women (5.19 ± 0.94) (46.1% of whom indicated a perception score of 6) than in men (4.08 ± 1.16; 8.3% indicated a perception score of 6).

Taking into consideration the perceived attributes, for all subjects, the taste intensity of Mirtamaro corresponded to the intensity of its bitterness. Therefore, in this subpopulation, women showed a higher Mirtamaro bitter taste intensity rating than men, confirming the results obtained for the classical bitter stimuli quinine and PROP. In general, most women perceived the odor/aroma of the bitter liqueur as very pleasant, while they indicated the high bitterness as unpleasant, whereas men perceived the Mirtamaro aroma as less pleasant than women and did not indicate the high bitterness as unpleasant.

In this subpopulation, significant differences (*p* < 0.01) were observed for the quinine taste intensity rating between women with a body weight ≤ 65 kg (3.38 ± 0.62) versus those with a body weight > 65 kg (2.40 ± 0.84); however no statistically significant differences emerged concerning body weight for PROP, Mirtamaro odor intensity, and taste intensity (Figure S4 Supporting Information).

Then, the correlation between the quinine taste intensity, PROP taste intensity, and Mirtamaro odor and taste dimensions (pleasantness, intensity, and familiarity) were calculated in men and women (Table 5).

Unless marked differences emerged between men and women, no significant correlations were found between quinine taste intensity, PROP taste intensity, and Mirtamaro taste bitterness perception (taste intensity) in either group.

Interestingly, strong correlations were found between the different Mirtamaro odor (OP, OI, OF) and taste dimensions (TP, TI, TF). Our data showed that women exhibited more correlations than men between different Mirtamaro odor and taste dimensions. Significant positive correlations were determined for OP/OF, OP/TI, OI/TI, OF/TI, and TP/TF in men, and OP/OF, OP/TP, OP/TF, OI/TI, OF/TP, OF/TF, and TP/TF in women (Table 5).

Mirtamaro odor intensity and taste intensity were strictly related both in men (r = 0.687, *p* < 0.05) and women (r = 0.508, *p* < 0.01), indicating that a high odor intensity rating corresponded to a high taste intensity rating. Moreover, strong correlations were found between odor pleasantness/familiarity and taste pleasantness/familiarity in both groups, indicating that familiarity with the bitter liqueur aroma and taste influenced their likability. Interestingly only women showed a strong correlation between odor pleasantness/taste pleasantness (r = 0.564, *p* < 0.01), indicating the contribution of Mirtamaro aroma in taste acceptance, and therefore a positive modulation of bitterness perception.

Furthermore, the multivariate linear regression analysis using a stepwise selection was performed using bitter liqueur taste pleasantness as the dependent variable and all other odor (OP, OI, OF) and taste (TI, TF) dimensions as independent variables in men and women (Table 6).

The multivariate linear regression analyses in men exhibited a significant association only between taste pleasantness and taste familiarity (TF) (Table 4) (F_(1,13_) = 7.371, *p* < 0.05). Instead, women showed significant associations between taste pleasantness and odor pleasantness (OP) and taste familiarity (TF) (F_(1,25)_ = 11.168, *p* < 0.01).

### 3.3. Main Volatile Compounds in the Aromatic Myrtle Herbal Liqueur (Mirtamaro)

To understand the potential contribution of aroma components on bitter taste perception/acceptance, the main volatile compounds of Mirtamaro liqueur were isolated by liquid–liquid extraction with *n*-hexane and analyzed by GC-FID/MS. The use of *n*-hexane as an extracting solvent allowed us to obtain non-polar flavor compounds [21,33].

**Figure 6:**
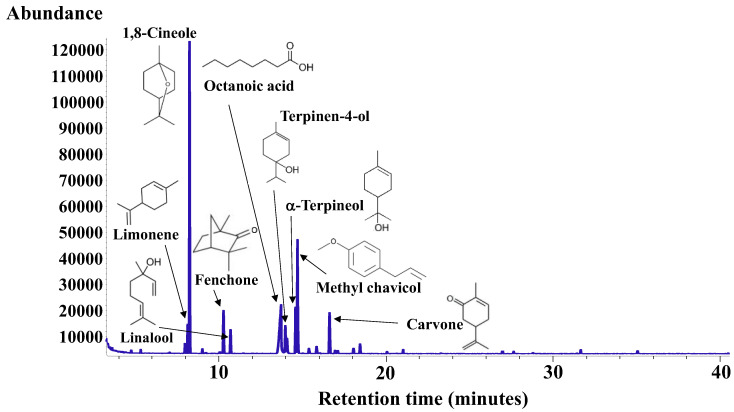
Chromatographic profiles by GC-FID of *n*-hexane extract obtained from the aromatic myrtle herbal liqueur (Mirtamaro). Chemical structures of the main volatile organic compounds are also reported.

Figure 6 shows the chromatographic profile by GC-FID analysis of Mirtamaro *n*-hexane extract, with the indication of the main identified volatile compounds.

The main volatile components of Mirtamaro, their percentages (% *w*/*w*), and their retention indices are reported in Table 7. The odor descriptions of Mirtamaro volatile components and literature references are also reported in Table 7.

Chemical analysis revealed that Mirtamaro was characterized by high amounts of oxygenated monoterpenes (fenchone, linalool, terpinen-4-ol, alpha-terpineol, and gamma-terpineol) and phenylpropanoids (methyl chavicol and (E)-anethole). However, the aromatic liqueur contained lower amounts of monoterpene hydrocarbons (alpha-thujene, *orto*-cymene, limonene) and other compound classes (octanoic acid). The main constituents of the extract were: 1,8-cineole (35.11%), methyl chavicol (estragole) (13.73%), octanoic acid (9.72%), alpha-terpineol (5.65%), fenchone (5.30%), and carvone (5.16%). Other minor compounds were: terpinen-4-ol (3.26%), limonene (3.10%), linalool (2.81%), gamma-terpineol (1.77%), (E)-anethole (0.87%), and alpha-thujene (0.26%).

## 4. Discussion

Bitterness is usually considered the most unpleasant taste by humans since it is associated with the bitter taste of poisonous compounds and our instinct is to reject them [1,2]. However, most of the natural bitter compounds found in herbs/plants have bioactive properties in the human organism [1,2]. Several herbs rich in bitter chemicals are often used in traditional Chinese and Ayurveda medicines, which are based on the cross-cultural belief that the bitterness of medicine is correlated with the desired medicinal activity [3]. A phytochemical bitter taste has been proposed as a better predictor of anti-inflammatory activity than the chemical class [5]. Several chemical functional groups (–NO_2_, =N–, –SH, –S–, –SO_3_H, –S–S, =C, =S) are often associated with bitter taste [1,2]. The Bitter Database (BitterDB) has gathered information on more than 1000 bitter compounds [1,40]. Several compounds can be perceived as bitter, such as ions, peptides, humulones, polyphenols (flavonoids, tannins), alkaloids, terpenes, iso-α-acids, higher alcohols, and glucosinolates [1,2]. Polyphenols are mainly responsible for the bitterness in fruits and vegetables [1,2].

Studies on bitterness perception have nutritional, nutraceutical, pharmaceutical, and health implications. Gustatory perception of the bitter taste seems to be related to an enhanced intake of dietary fat and to a tendency to develop obesity [17]. Bitter-taste sensitivity greatly changes among individuals due to the genetic variability of bitter-taste receptor TAS2Rs (polymorphisms) [1,2,7,9,17]. Moreover, a significant decrease in bitter perception with increasing age has been reported [17,18,19]. Studies devoted to investigating the influence of sex on bitter taste acuity demonstrated that women have better bitterness perception than men; however, data are very contrasting [7,17,18,19]. The influence of sex in bitter taste perception should be taken into account for potential applications within the new precision nutrition/precision medicine framework [18,20].

In the first part of this study, we analyzed the influence of sex on the intensity rating of the four basic tastes: sweet, salty, bitter, and sour (separately and jointly in a “total taste score”) in a healthy population. Our results showed that women perceived bitter taste (by challenging subjects with different quinine concentrations), as well as sweet, salt, and total taste, significantly more intensely than men.

According to our results, a greater taste perception in women has been reported for several taste qualities [17,18,41], although the results are not always consistent. Several studies have demonstrated that women perceive taste significantly more intensely than men and score better in taste identification tests [17,18,41]; however many other studies found no relationship between sex and gustatory function [41]. A large epidemiological study reported statistically significant differences between women and men for bitter (quinine), sweet, salty, and sour tastes, and women perceived each taste significantly more intensely than men [41]. Barragan et al. [18] found that women consistently have a greater perception of bitter (PROP), sweet, salty, and sour than men, except for umami. Previous studies evidenced that bitter taste perception can be different between men and women, though both have the same gene expression about bitterness, and women have a better perception of bitterness than men and a strong tendency to be supertasters [17]. However, no statistically significant differences were observed between males and females related to the perception of the bitter taste of the steroid prednisolone [7]. Moreover, a study conducted to determine the influence of age and sex on the taste functions of healthy Taiwanese subjects showed that all individual tastes were rated as similarly intense regardless of sex [19]. The mechanism through which sex/gender affects the sense of taste is not exactly known, but it may be related to hormonal influences on taste, and a complex association between hormones and chemoreceptor functions has been suggested to exist in women [17].

Regarding bitterness perception in women, our data showed a significant negative association between bitter taste intensity versus weight, indicating a decrease in bitter perception with a weight increase. Significant lower bitter taste scores were measured in women with a body weight > 65 kg (BMI mean value > 28 kg/m^2^) versus those with a body weight ≤ 60 kg (BMI < 28 kg/m^2^). Previous studies suggested that the sensitivity to bitterness is related to body weight and reported an inverse correlation between the perception of PROP bitterness and BMI [17].

Strategies employed to reduce/mask bitterness include the use of other taste stimuli (such as sucrose and sodium chloride) or bitter modifiers/blockers, compounds that affect bitter perception by the modulation of the human bitter taste receptors, complexation/encapsulation of bitterants, or the formation of a physical barrier between bitterant and taste receptors [42,43]. In the last years, a remarkable increase has been observed in the dietary use of aromatic herbs and spices for their ability to provide complex flavor to food products and positively affect human health [21,25,44]. Aromatic plants, used as flavor enhancers, provide phytochemicals, essential oils, proteins, vitamins, minerals, and fiber, greatly contributing to the promotion of health due to their antioxidant and anticancer activity and capacity to prevent cardiovascular/neurodegenerative diseases [21,44]. The use of aromatic plants, spices, and essential oils to prepare beverages dates back to ancient Mediterranean history [11]. Herbal liqueurs are descendants of former cordials, medicinal plants, alcoholic extracts, or elixirs that were believed to manifest curative properties [10]. The bitter taste is also constantly dropping its popularity among consumers [4] and traditional bitter liqueurs are also consumed for their supposed pharmacological activity (digestive, anti-inflammatory, and antioxidant properties) [5,10,11,12,14]. Italy seems to produce the largest number and widest variety of bitter, herbal liqueurs traditionally consumed as aperitifs or digestives, usually called “Amari”, or, literally, “bitters” [11]. Plants belonging to *Gentiana*, *Artemisia*, and *Achillea* spp. are largely used for conferring bitterness to aromatic bitter liqueurs [11]. The most popular bitter botanical used in alcoholic beverages is gentian (*Gentiana lutea*) (mainly roots), a plant with important healthy properties (digestive, stimulating the appetite, curing indigestion, and easing constipation) [45,46].

Myrtle (*M. communis* L.) is an aromatic plant endemic in the Mediterranean area and has long been used by locals in traditional medicine (for treating several common diseases, including gastrointestinal, urinary, and skin disorders) and for its culinary properties. Myrtle berries, leaves, seeds, and essential oils are currently widely employed in the food, cosmetic, and pharmaceutical industries for their antioxidant and anti-inflammatory properties [11,28,29]. Myrtle berries and leaves are amply used for food aromatization, and to prepare, by hydro-alcoholic maceration, a typical liqueur (“Mirto”) that is very popular, especially on Sardinia Island [11,28]. Mirtamaro is a recent after-meal liqueur, characterized by a bitter flavor, obtained by the maceration of myrtle leaves and berries together with a complex (secret) mixture of aromatic Mediterranean herbs and plants as flavoring/bittering ingredients. In the second part of this study, we evaluated, in a group of 40 healthy subjects, sex differences in the sensory perception (odor and taste) of this aromatic myrtle bitter liqueur and explored the role of volatiles (aroma) from aromatic herbs/plants in the bitter taste perception/acceptance.

All participants perceived the odor of the aromatic bitter liqueur as very pleasant and familiar; however, women perceived Mirtamaro odor more intensely than men and were able to discriminate some aroma components. Many studies report superior female performance on tests of odor identification [47]. All subjects perceived the Mirtamaro taste as less pleasant and familiar than the odor. Moreover, both men and women indicated bitterness as the main taste modality of the aromatic liqueur. In many cases, participants indicated an initial sweet taste perception followed by a strong bitter taste. It is well known that the time necessary for bitterness perception in the mouth is greater than for other tastes [2]. Women perceived the Mirtamaro bitter taste more intensely than men, confirming the influence of sex on the bitterness perception. The higher perception of bitterness in women made the bitter liqueur taste more unpleasant than for men. In this group of participants, women also perceived the bitter taste of PROP, as well as quinine, significantly more intensely than men.

Significant positive correlations emerged between bitter liqueur odor pleasantness/odor familiarity and taste pleasantness/taste familiarity both in men and women. Many studies demonstrated a positive correlation between odor familiarity and pleasantness, which represents a consistent result in olfactory research [48]. Moreover, it is well demonstrated that the habitual consumption (familiarity) of a food/liqueur raises its acceptability [27,49]. Interestingly, a positive correlation was found between Mirtamaro odor intensity/taste intensity in both groups. The perception of food flavor affecting food choice derives from the integration of olfactory/gustatory information [27,49]. Additional significant positive correlations emerged between bitter liqueur odor pleasantness/taste pleasantness, odor pleasantness/taste familiarity, odor familiarity/taste pleasantness, and odor familiarity/taste familiarity in women, indicating a complex integration between olfactory and gustatory functions. Interestingly, the strong positive correlation between odor pleasantness and taste pleasantness measured only in women possibly indicated the contribution of Mirtamaro aroma in taste acceptance, and therefore a positive modulation of bitterness acceptance due to pleasant volatile aromatic compounds.

Volatile organic flavor compounds are responsible for food/liqueur aroma/odor and are perceived through the smell sensory organs of the nasal cavity (ortho-nasal smell) [27,50]. Flavor involves the combination of gustative perception of soluble and non-volatile compounds (basic tastes), volatile compounds perceived through retro-nasal olfaction (aroma), and chemical sensations through the trigeminal nerve [21,50]. A complex relationship exists between the presence of specific configurations of volatile organic compounds in food and drink products and multisensory flavor perception [51]. The bitterness properties of alcoholic beverages primarily result from the various raw materials, unique techniques, and interactions of various flavor compounds [2]. Aroma and taste activate the central cognitive pathway to generate flavor perception [2,22,50]. Previous studies evidenced that odor–taste interactions can result in cross-modal summation [2,22] and that bitterness is cognitively related to aromas [2]. It has been reported that the rich aromatic substances in alcoholic beverages may influence bitterness perception [2].

More than twenty aromatic herbs/plants (among others gentian, *Citrus* fruits, licorice, helichrysum, and fennel) are mixed (according to a secret recipe and the formula was not publicized) to myrtle leaves/berries to obtain the right balance of bitter, balsamic, spicy, and citrus flavors of Mirtamaro. The root of gentian is used for conferring bitterness. The main volatile compounds of Mirtamaro liqueur were isolated by liquid–liquid extraction with *n*-hexane and analyzed by GC-FID/MS. *n*-Hexane has previously been reported as a proper solvent for the extraction of volatile compounds in honey and flavored sea salts without the extraction of polar components (such as sugars, salt, and water) [21,33]. 1,8-Cineole, methyl chavicol (estragole), octanoic acid, alpha-terpineol, fenchone, and carvone emerged as the main Mirtamaro aroma components, while terpinen-4-ol, limonene, linalool, gamma-terpineol, (E)-anethole, *orto*-cymene, and alpha-thujene represented minor components. Compounds such as 1,8-cineole, alpha-thujene, linalool, *orto*-cymene, limonene, terpinen-4-ol, and alpha-terpineol are typical components of myrtle berries and leaves and are also responsible for their characteristic aroma and taste [21,29]. Fenchone, estragole, and (E)-anethole are the compounds that characterize the aroma of wild fennel [52]. The aromatic terpene oxide eucalyptol (1,8-cineole) and the phenylpropene methyl chavicol (estragole) represented the most abundant volatile compounds in Mirtamaro, accounting for approximately 49% of volatile compounds. Both compounds possibly contributed to the odor pleasantness of the bitter liqueur, being characterized by a pleasant odor (descriptors: camphor-like, citrus, herbaceous, and fruity for 1,8-cineole [38,39,53]; sweet, phenolic, fennel, anise, spicy, green, and herbal for estragole [38,39,53]).

Phenolic compounds, anthocyanins, and essential oil are the most important phytochemicals in myrtle berries and leaves [11,28]. Previous studies showed that myrtle liqueur is composed of arabinoside derivatives, flavonols, flavanols, hydroxybenzoic acids, and anthocyanins with malvidin-3-*O*-glucoside, petunidin-3-*O*-glucoside, and delphinidin-3-*O*-glucoside as the most representative ones [11,28]. Phenolic compounds have a great influence on the final bitter taste due to their bitterness and astringency notes [2,14,54]. Gentian roots are a high source of bitter molecules such as the secoiridoid glycosides amarogentin and gentiopicroside [45,46]. Previous studies indicated the non-volatile metabolites gentisin, isogentisin, swertiamarin, sweroside, gentiopicroside, loganic acid, and amarogentin as the most abundant compounds in gentian liqueurs produced by simple maceration of the dried or fresh gentian roots in spirits [45]. Therefore, Mirtamaro flavor perception is the result of a multisensory interaction due to the presence of multitudinous flavors and bitter compounds.

Therefore, volatile compounds measured in *n*-hexane extracts and other non-volatile polar components present in a hydro-alcoholic solution could contribute to the taste/flavor of Mirtamaro and bitterness perception. Non-volatile polar compounds of Mirtamaro may modulate the taste perception on taste buds in the tongue, whereas aromatic compounds present in bitter liqueur, liberated in the mouth, may be responsible for the flavor attributes indicated by the untrained panelists through retro-nasal olfaction. However, the exact sensory impact of analyzed volatile compounds was difficult to predict due to the wide range of aroma qualities associated with all identified terpenes [13,53].

Differences between men and women have been reported in the functional neural connectivity of the gustatory network as modulated by the perception of sweet and bitter tastes [55]. Taste acceptance is generally guided by perceived pleasure and reward, which have a strong impact on taste preference and feeding behavior [56]. Our results showed that women exhibited a greater ability than men to perceive Mirtamaro aroma (odor intensity dimension) and bitter taste (taste intensity dimension). A high rating in aromatic liqueur odor intensity corresponded to a high rating in taste intensity perception in both groups; however, our data did not demonstrate an evident modulatory effect of aromatic compounds in potentiating or reducing bitterness intensity perception. Probably in women, the odor/aroma pleasantness, conferred by the presence of the blend of aromatic herbs/plants, ameliorated the taste pleasantness and therefore the acceptance of the liqueur’s bitter taste.

## 5. Conclusions

Bitterness greatly influences the acceptance of health-promoting bitter foods and natural bitter compounds; therefore, studies on bitter taste perception have important nutritional and pharmaceutical implications. Our results showed that women exhibited a higher bitter taste intensity rating than men for common bitter stimuli such as quinine and PROP. Moreover, women showed higher ratings than men in bitterness (taste intensity dimension) perception of the aromatic myrtle bitter liqueur. There is currently a great interest in the incorporation of sex-dependent differences into health research and in the integration of sex-based analysis into study design for nutrition research and pharmaceutical product development. The results of the present study represent a significant contribution to the limited existing literature on the occurrence of sex differences in bitter taste perception, with potential applications in the field of precision nutrition and medicine.

Aromatic herbs and spices are an important dietary source of bioactive and health-promoting compounds and are traditionally used to impart characteristic flavorings to food products. In this study, women showed higher ratings than men in aroma (odor intensity dimension) perception of the aromatic myrtle bitter liqueur, with a superior capacity to perceive/describe bitter liqueur sensory attributes. A statistically significant positive relationship was observed in women between Mirtamaro odor pleasantness and taste pleasantness, indicating a contribution of volatile compounds from aromatic plants to bitter taste pleasantness/acceptability. The results of the present study provided new evidence on the traditional role of health-beneficial aromatic herbs and spices as flavor-enhancing ingredients, qualifying their use as an important strategy for modulating bitterness perception/acceptance specifically in women. The use of blends of herbs and spices characterized by a suitable pleasant flavor may be a promising natural strategy to ameliorate the acceptance of health-beneficial bitter foods and natural bitter compounds for nutritional, nutraceutical, and pharmaceutical applications.

## Figures and Tables

**Table 1:** Demographic and clinical features, expressed as mean ± standard deviation, of total subjects, men, and women.

Parameters	Total Subjects (*n* = 231)	Men (*n* = 78)	Women (*n* = 153)
Age	35.8 ± 15.6	35.9 ± 15.8	35.7 ± 16.0
Weight (kg)	64.8 ± 15.0	75.8 ± 12.6	59.2 ± 12.9 ***
Height (m)	1.6 ± 0.1	1.7 ± 0.1	1.6 ± 0.1
BMI	24.0 ± 6.4	26.3 ± 8.6	22.8 ± 4.4 **
OThr	7.3 ± 4.7	7.5 ± 5.0	7.1 ± 4.5
ODi	11.6 ± 2.2	11.4 ± 2.3	11.7 ± 2.2
OId	12.9 ± 1.8	12.7 ± 1.7	13.0 ± 1.9
TDI score	31.7 ± 6.4	31.5 ± 6.6	31.8 ± 6.4

Legend: BMI = body mass index; OThr = odor threshold; ODi = odor discrimination; OId = odor identification; TDI score = threshold, discrimination, and identification score. *** = *p* < 0.001; ** = *p* < 0.001 for men versus women (Student’s unpaired *t*-test with Welch’s correction).

**Table 2:** Pearson’s correlations (r) and significance (*p*) calculated between bitter (quinine) taste intensity rating versus other parameters in total subjects (231), men (78), and women (153).

Parameters	Bitter Intensity					
	Total Subjects		Men		Women	
	r	*p*	r	*p*	r	*p*
Sex	−0.187	*p* < 0.01	-	-	-	-
Age	0.003	*p* > 0.05	0.060	*p* > 0.05	−0.034	*p* > 0.05
Weight	−0.164	*p* < 0.05	0.125	*p* > 0.05	−0.217	*p* < 0.01
BMI	−0.063	*p* < 0.05	0.116	*p* > 0.05	−0.191	*p* < 0.05
OTr	−0.029	*p* > 0.05	−0.050	*p* > 0.05	0.000	*p* > 0.05
ODi	−0.034	*p* > 0.05	−0.059	*p* > 0.05	−0.044	*p* > 0.05
OId	0.030	*p* > 0.05	−0.038	*p* > 0.05	0.047	*p* > 0.05
TDI score	−0.024	*p* > 0.05	−0.068	*p* > 0.05	−0.001	*p* > 0.05
Sweet	0.233	*p* < 0.01	0.283	*p* < 0.05	0.132	*p* > 0.05
Salty	0.117	*p* > 0.05	0.028	*p* > 0.05	0.144	*p* > 0.05
Sour	0.103	*p* > 0.05	0.173	*p* > 0.05	0.034	*p* > 0.05
Total taste	0.651	*p* < 0.001	0.695	*p* < 0.001	0.585	*p* < 0.001

Abbreviation: BMI = body mass index; OThr = odor threshold; ODi = odor discrimination; OId = odor identification; TDI score = threshold, discrimination, and identification score.

**Table 3:** Multivariate linear regression analyses in total subjects, men, and women using bitter (quinine) taste intensity score as the dependent variable.

Parameters	Unstandardized Coefficients		Standard Coefficients		
	B	Std Error	β	t	Significance
Total subjects					
Age	−0.244	0.177	−0.104	−1.373	*p* > 0.05
Sex	−0.437	0.152	−0.187	−2.873	*p* < 0.01
Sweet	0.255	0.082	0.201	3.094	*p* < 0.01
Weight	−0.006	0.006	0.281	3.094	*p* > 0.05
Men					
Age	0.004	0.009	0.074	0.661	*p* > 0.05
Weight	0.011	0.012	0.110	0.989	*p* > 0.05
Sweet	0.372	0.143	0.289	2.603	*p* < 0.05
Women					
Age	0.001	0.005	0.021	0.261	*p* > 0.05
Weight	−0.016	0.006	−0.211	−2.626	*p* < 0.01
Sweet	0.149	0.101	0.119	1.471	*p* > 0.05

Legend: B = unstandardized coefficient for each predictor variable; β = standardized coefficient, which gives a measure of the variable contribution; t = t-values, which indicate whether the predictor’s regression coefficient is significant.

**Table 4:** The subjective sensory evaluation of the odor (aroma) and taste (flavor) of myrtle herbal liqueur (Mirtamaro) in men (*n* = 14) and women (*n* = 26).

Sex	Sensory Input	Sensory Perceived Attributes
Men	Odor	Myrtle; herbs; liqueur; licorice; bitter; natural essences.
	Taste	Bitter; very bitter, and a bit sour; sweet at the beginning, then bitter; myrtle note.
Women	Odor	Liqueur; bitter liqueur; myrtle; alcohol; bitter; very bitter; chinotto; licorice; juniper; berries; orange; woody; spicy; rum; nicotine; coffee; sambuca; medication; pungent.
	Taste	Bitter; alcohol; very bitter; myrtle note; *Citrus* note; pungent; chinotto note; slightly bitter aftertaste; mint aftertaste; sweet at the beginning, then very bitter; woody note; quinine; whiskey; sweet; caramel aftertaste.

**Table 5:** Pearson’s correlations (r) and significance (*p*) calculated between quinine taste intensity, PROP taste intensity, Mirtamaro odor (PO, IO, FO) and taste (TP, TI, TF) dimensions in all subjects (40), men (14), and women (26).

Data	Quinine	PROP	OP	OI	OF	TP	TI	TF
Quinine	1							
PROP	0.214	1						
OP	−0.172	−0.094	1					
OI	−0.469	−0.113	0.347	1				
OF	−0.021	−0.269	0.777 **	0.434	1			
TP	−0.158	−0.237	−0.138	0.093	−0.012	1		
TI	−0.140	−0.316	0.562 *	0.687 *	0.606 *	0.055	1	
TF	−0.064	−0.625 *	−0.168	0.351	0.024	0.651 *	0.320	1
Quinine	1							
PROP	−0.305	1						
OP	0.176	−0.234	1					
OI	0.159	0.117	0.017	1				
OF	0.147	−0.283	0.735 ***	0.160	1			
TP	0.303	−0.304	0.564 **	0.041	0.542 **	1		
TI	0.201	0.244	−0.142	0.508 **	−0.064	−0.200	1	
TF	0.175	−0.319	0.518 **	0.149	0.572 **	0.659 ***	−0.072	1

Abbreviation: PROP = 6-n-propylthiouracil; OP = odor pleasantness; OI = odor intensity; OF = odor familiarity; TP = taste pleasantness; TI = taste intensity; TF = taste familiarity. * = *p* < 0.05, ** = *p* < 0.01, *** = *p* < 0.001.

**Table 6:** Multivariate linear regression analyses in men (*n* = 14) and women (*n* = 26) using Mirtamaro taste pleasantness (TP) as the dependent variable.

Parameters	Unstandardized Coefficients		Standard Coefficients		
	B	Std Error	β	t	Significance
Men					
OP	−0.029	−0.038	0.972	−0.113	*p* > 0.05
OI	−0.154	−0.152	−0.187	−0.582	*p* > 0.05
OF	−0.028	−0.036	0.999	−0.109	*p* > 0.05
TI	−0.171	−0.036	0.898	−0.654	*p* > 0.05
TF	0.796	0.293	0.651	2.715	*p* < 0.05
Women					
OP	0.562	0.168	0.564	3.342	*p* < 0.01
OI	−0.040	−0.265	0.794	−0.056	*p* > 0.05
OF	0.278	1.125	0.272	0.228	*p* > 0.05
TI	−0.122	−0.709	0.486	−0.146	*p* > 0.05
TF	0.436	0.150	0.502	2.915	*p* < 0.01

Legend: OP = odor pleasantness; OI = odor intensity; OF = odor familiarity; TP = taste pleasantness; TI = taste intensity; TF = taste familiarity. B = unstandardized coefficient for each predictor variable; β = standardized coefficient, which gives a measure of the variable contribution; t = t-values, which indicate whether the predictor’s regression coefficient is significant.

**Table 7:** Chemical composition (expressed as % *w*/*w* of total volatiles) by GC-FID of *n*-hexane extract obtained from the aromatic myrtle herbal liqueur (Mirtamaro), the retention time (RT), retention index (RI), retention index from literature (RI_Lit_), and odor description of identified compounds.

RT	RI	RI_Lit_	Compound	% *w*/*w*	Identification ^a^	Odor Description ^b^
5.475	940	930	alpha-thujene	0.26 ± 0.02	RI,MS	Woody, green, herbal [38]
8.120	1027	1026	*orto*-cymene	1.12 ± 0.23	RI,MS	-
8.281	1032	1029	Limonene	3.10 ± 0.39	RI,MS	Citrus-like, orange, fresh, sweet [38,39]
8.393	1035	1031	1,8-Cineole	35.11 ± 2.81	RI,MS	Eucalyptus, camphor-like [38,39]
10.429	1089	1086	Fenchone	5.30 ± 0.38	RI,MS	Camphor-like, fresh, woody [38,39]
10.845	1098	1096	Linalool	2.81 ± 0.03	RI,MS	Floral, sweet, spicy, woody, green [38,39]
13.803	1172	1171	Octanoic acid	9.72 ± 2.87	RI,MS	Faint, fruity-acid [38]
14.124	1179	1177	Terpinen-4-ol	3.26 ± 0.19	RI,MS	Pine [38]
14.733	1192	1188	alpha-terpineol	5.65 ± 0.26	RI,MS	Floral, lilac [38]
14.853	1195	1196	Methyl chavicol	13.73 ± 1.85	RI,MS	Spicy, green, herbal, fennel, anise [38,39]
15.158	1201	1199	gamma-terpineol	1.77 ± 0.05	RI,MS	Lilac [38]
16.769	1242	1243	Carvone	5.16 ± 0.32	RI,MS	Spicy, mint, green [39]
18.604	1283	1284	(E)-Anethole	0.87 ± 0.34	RI,MS	Anise, licorice, medicinal [38,39]
-	-	-	N.I.	12.17 ± 2.70		-

Legend: N.I. = not identified. RI = retention index determined on a HP-5ms fused silica column relative to a series of n-alkanes; RI (Litt) = retention index reported from Adams libraries [36]. ^a^ Compounds were identified by comparing their mass spectra (MS) and retention indices (RI) with those reported in NIST05 [35] and Adams [36] libraries. ^b^ Obtained from literature references [38,39].

## Data Availability

The datasets generated and analyzed during the current study are available from the corresponding author on reasonable request.

## Supplementary Materials

The following supporting information can be downloaded at: https://www.mdpi.com/article/10.3390/nu15092030/s1.
