# Supplementary material for: Sex Differences in the Bitterness Perception of an Aromatic Myrtle Bitter Liqueur and Bitter Compounds
Source: Nutrients. 2023 Apr 23;15(9):2030. doi: 10.3390/nu15092030 (PMC10181268; doi:10.3390/nu15092030)
Supplement: Supplementary file 1 [file nutrients-15-02030-s001.zip › nutrients-2312764-supplementary.pdf]

Article

Sex Differences in the Bitterness Perception of an Aromatic Myrtle Bitter-Liqueur and Bitter Compounds

Antonella Rosa[1,*], Ilenia Pinna[1], Alessandra Piras[2], Silvia Porcedda[2] and Carla Masala[1]

[1] Department of Biomedical Sciences, University of Cagliari, Cittadella Universitaria, SS 554, Km 4.5, 09042 Monserrato, Cagliari, Italy; anrosa@unica.it (A.R.); ilenia.pinna.1994@gmail.com (I.P.); cmasala@unica.it (C.M.)

[2] Department of Chemical and Geological Sciences, University of Cagliari, Cittadella Universitaria, SS 554, Km 4.5, 09042, Monserrato, Italy; apiras@unica.it (A.P.); porcedda@unica.it (S.P.)

* Correspondence: anrosa@unica.it; Tel.: 0039 0706754124

Table of Contents

Table S1: Demographic and clinical features of total subjects, men, and women enrolled for Mirtamaro sensory assessment.

Figure S1: Mean values ± standard deviation (SD) of bitter (quinine) taste scores measured in men and women separated into three different age-groups: 18-36 years, 37-50 years, and 51-85 years.

Figure S2: Mean values ± standard deviation (SD) of sweet, salty, and sour taste scores measured in women with a body weight ≤ 65 kg and > 65 kg.

Figure S3: Values ± standard deviation (SD) of sweet, salty, and sour taste scores measured for total subjects, men and women enrolled for Mirtamaro sensory assessment.

Figure S4: Mean values ± standard deviation (SD) of quinine and PROP bitter taste scores, Mirtamaro odor intensity (OI) and taste intensity (TI) measured in women with a body weight ≤ 65 kg and > 65 kg.

Citation:

https://doi.org/10.3390/xxxxx

Academic Editor(s):

Received: date
Accepted: date
Published: date

Publisher's Note: MDPI stays neutral with regard to jurisdictional claims in published maps and institutional affiliations.

Copyright: © 2023 by the authors. Submitted for possible open access publication under the terms and conditions of the Creative Commons Attribution (CC BY) license (https://creativecommons.org/licenses/by/4.0/).

Table S1. Demographic and clinical features of total subjects, men, and women enrolled for Mirtamaro sensory assessment.

**Parameters**	**Total subjects** (*n* = 40)	**Men** (*n* = 14)	**Women** (*n* = 26)
Mean age	48.9 ± 19.8	39.2 ± 22.1	48.6 ± 18.4
Weight (kg)	64.8 ± 16.1	78.8 ± 12.2	64.2 ± 15.8**
Height (m)	1.6 ± 0.1	1.7 ± 0.1	1.6 ± 0.1
BMI	25.8 ± 5.3	26.9 ± 4.6	25.3 ± 5.6
OThr	8.5 ± 5.4	8.7 ± 5.4	8.4 ± 5.4
ODi	11.4 ± 2.5	11.2 ± 2.0	11.5 ± 2.7
OId	13.1 ± 2.0	12.2 ± 2.0	13.5 ± 1.9
TDI score	32.9 ± 6.4	32.2 ± 7.0	33.3 ± 8.4

Legend: W: women; M: men; BMI = body mass index; OTr = odor threshold; ODi = odor discrimination; OId = odor identification; TDI score = threshold, discrimination, and identification score. ** = $p < 0.01$ for men *versus* women (Student's unpaired t-test with Welch's correction).

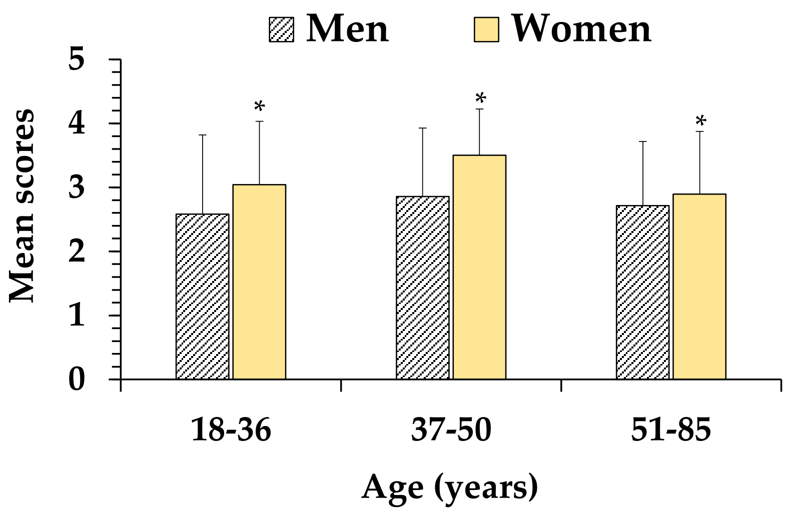

Figure S1. Mean values ± standard deviation (SD) of bitter (quinine) taste scores measured in men (n = 78) and women (n = 153) separated into three different age-groups: 18-36 years (*n* = 50 for men and n = 91 for women), 37-50 years (*n* = 10 for men and *n* = 24 for women), and 51-85 years (*n* = 18 for men and *n* = 38 for women). * = $p < 0.05$ (Student's unpaired t-test with Welch's correction) for men *versus* women.

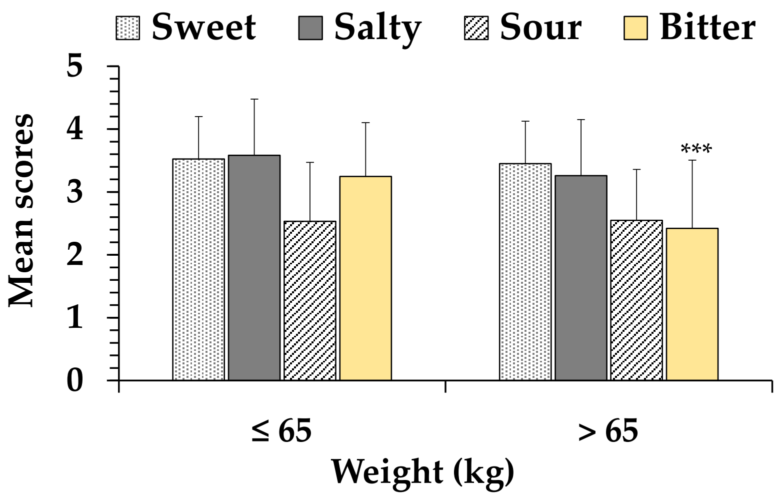

Figure S2. Mean values ± standard deviation (SD) of sweet, salty, and sour taste scores measured in women ($n = 153$) with a body weight ≤ 65 kg ($n = 122$) and > 65 kg ($n = 31$). *** = $p < 0.001$ (Student's unpaired t-test with Welch's correction) between subjects with a body weight > 65 kg *versus* those with a body weight ≤ 65 kg.

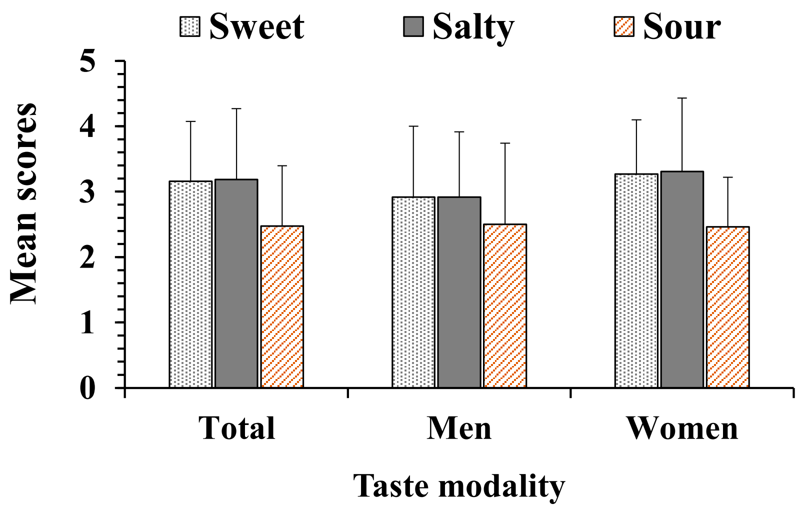

Figure S3. Mean values ± standard deviation (SD) of sweet, salty, and sour taste scores measured in total subjects (Total, n = 40), men (*n* = 14), and women (*n* = 26) enrolled for Mirtamaro sensory assessment.

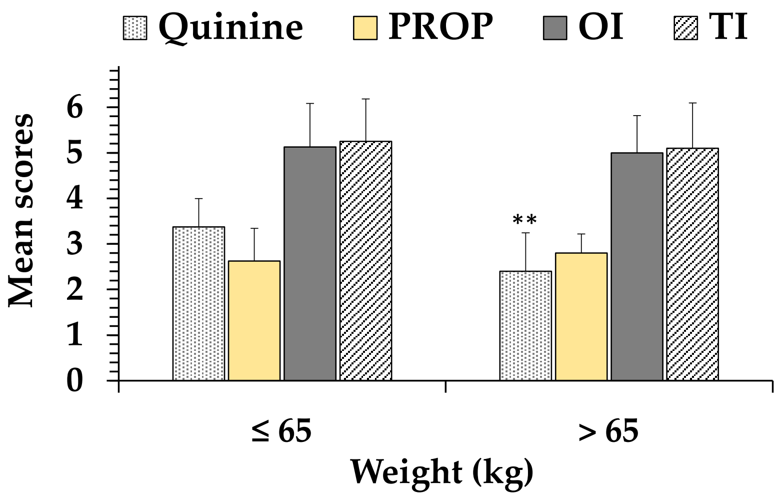

Figure S4. Mean values ± standard deviation (SD) of quinine and PROP bitter taste scores, Mirtamaro odor intensity (OI) and taste intensity (TI) measured in women (n = 26) with a body weight ≤ 65 kg (n = 16) and > 65 kg (n = 10). ** = $p < 0.01$ (Student's unpaired t-test with Welch's correction) between subjects with a body weight > 65 kg *versus* those with a body weight ≤ 65 kg.
